# Genome-Wide Association Study Identifies Resistance Loci for Bacterial Blight in a Collection of Asian Temperate *Japonica* Rice Germplasm

**DOI:** 10.3390/ijms24108810

**Published:** 2023-05-16

**Authors:** Jianmin Li, Xiaorong Shi, Chunchao Wang, Quanlin Li, Jialing Lu, Dan Zeng, Junping Xie, Yingyao Shi, Wenxue Zhai, Yongli Zhou

**Affiliations:** 1 National Key Facility for Crop Gene Resources and Genetic Improvement/Institute of Crop Sciences, Chinese Academy of Agricultural Sciences, Beijing 100081, China; 2 National Nanfan Research Institute (Sanya), Chinese Academy of Agricultural Sciences, Sanya 572024, China; 3 College of Agronomy, Anhui Agricultural University, Hefei 230036, China; 4 Institute of Genetics and Developmental Biological, Chinese Academy of Sciences, No. 1 Beichen West Road, Chaoyang District, Beijing 100101, China

**Keywords:** rice bacterial blight, GWAS, QTL, haplotype analysis

## Abstract

Growing resistant rice cultivars is the most effective strategy to control bacterial blight (BB), a devastating disease caused by *Xanthomonas oryzae* pv. *oryzae* (*Xoo*). Screening resistant germplasm and identifying resistance (*R*) genes are prerequisites for breeding resistant rice cultivars. We conducted a genome-wide association study (GWAS) to detect quantitative trait loci (QTL) associated with BB resistance using 359 East Asian temperate *Japonica* accessions inoculated with two Chinese *Xoo* strains (KS6-6 and GV) and one Philippine *Xoo* strain (PXO99^A^). Based on the 55K SNPs Array dataset of the 359 *Japonica* accessions, eight QTL were identified on rice chromosomes 1, 2, 4, 10, and 11. Four of the QTL coincided with previously reported QTL, and four were novel loci. Six *R* genes were localized in the *qBBV-11.1*, *qBBV-11.2*, and *qBBV-11.3* loci on chromosome 11 in this *Japonica* collection. Haplotype analysis revealed candidate genes associated with BB resistance in each QTL. Notably, *LOC_Os11g47290* in *qBBV-11.3*, encoding a leucine-rich repeat receptor-like kinase, was a candidate gene associated with resistance to the virulent strain GV. Knockout mutants of Nipponbare with the susceptible haplotype of *LOC_Os11g47290* exhibited significantly improved BB resistance. These results will be useful for cloning BB resistance genes and breeding resistant rice cultivars.

## 1. Introduction

Rice bacterial blight (BB) caused by *Xanthomonas oryzae* pv. *oryzae* (*Xoo*) is one of the most serious and widespread bacterial diseases. In Asian countries, there are frequent BB outbreaks that threaten rice yield [1,2]. Breeding and growing resistant cultivars harboring resistance (*R*) genes is the most effective, economical, and environmentally friendly strategy for controlling this disease in rice production [3,4]. It is therefore important to find new genes associated with BB resistance for successful crop improvement.

Since *Xa1* was identified in the 1960s [5], 47 *R* genes (*Xa*-genes) and some quantitative trait loci (QTL) conferring BB resistance have been identified in wild and cultivated rice and artificial mutants [6,7]. Of them, *Xa21*, *Xa23*, *Xa27*, *Xa29*, *Xa30(t)*, *Xa33*, *Xa35(t)*, *Xa38*, and *xa41(t)* were identified in wild rice species, and *xa15*, *xa19*, *xa20*, *xa42*, and *xa46(t)* were identified in artificial resistant mutants. More than 20 genes were found in cultivated rice, and most of them were identified in *indica* varieties. So far, 16 genes (or alleles) have been cloned, including *Xa1* and its alleles *Xa2/Xa31(t)*, *Xa14*, and *Xa45(t)*, *Xa3/Xa26*, *Xa4*, *xa5*, *Xa7*, *Xa10*, *xa13*, *Xa21*, *Xa23*, *xa25*, *Xa27*, *xa41(t)*, and *Xa47* [7]. However, among the identified *Xa*-genes, only *Xa4*, *Xa21*, *Xa23*, and *Xa39* with broad resistance spectra have been widely used in rice breeding programs in China [8,9]. The narrow genetic basis of BB resistance in rice cultivars has resulted in a dramatic change in the pathogen population and the subsequent emergence of virulent strains that can breakdown the resistance mediated by *Xa4*, *Xa21*, and *Xa23* in South China [10,11]. Therefore, it is necessary to identify novel genes/QTL conferring BB resistance and to explore new strategies to improve the BB resistance of rice.

With the rapid development of genome sequencing technologies, genome-wide association study (GWAS) provides a strategy for discovering favorable alleles in crops based on high density SNP genotypes and accurate phenotypic data for crop germplasm resources [12,13,14,15]. In recent years, diverse rice germplasm populations have been used to identify QTL and genes associated with BB resistance through GWAS. For example, seven QTL were closely linked with the known *R* genes *Xa4*, *xa5*, *Xa7*, *xa13*, *Xa14*, *Xa21*, *xa25*, and novel QTL were found on chromosomes 6, 9, 11, and 12 in 285 rice accessions [16], and 12 genomic regions associated with BB resistance were identified in 172 *indica* rice accessions [17]. A major QTL on chromosome 11 in a MAGIC population was identified and designated as *Xa43(t)* [6]. Recently, the sequence data for the 3000 Rice Genomes Project (3K RGP) provides the platform for large-scale discovery of genetic variation associated with important traits for breeding applications [18,19]. *Xa*-genes and QTL associated with BB resistance were detected, in different panels with diverse subgroups of 3K RGP, and the resistant alleles of *LOC_Os11g46250* and *LOC_Os11g46890* were further demonstrated to improve BB resistance in rice [11,20,21].

In the past, genetic manipulation of susceptible genes has been shown to confer disease resistance in various economically important crops. Recent studies have accomplished this task using new genome editing tools [22]. The development of the RNA guided CRISPR/Cas9 system provides an efficient method of choice for genome editing because of its simplicity, ease of performing, and versatility [23]. Much significant progress has been made in resistance improvement through genome editing mediated by CRISPR/Cas9 in rice [24,25,26,27].

As the excellent eating quality of *japonica* to the growing demands of consumers, the rice planting area in the temperate regions of China have increased substantially over the past few decades [28]. However, up to now, there are few reports on the exploitation of BB resistance genes and QTL in *japonica* rice germplasm panels. In this study, the BB resistance of 359 *Japonica* rice accessions was evaluated after inoculation with two Chinese representative *Xoo* strains and one Philippine *Xoo* strain. Through GWAS, eight genetic loci associated with BB resistance were identified on the basis of 55K single nucleotide polymorphism (SNP) genotypes of this *Japonica* rice collection and their phenotypes after inoculation with the three *Xoo* strains. The knockout mutants of Nipponbare harboring the susceptible allele of *LOC_Os11g47290* showed significantly increased BB resistance. These results provide useful information for cloning QTL associated with BB resistance and for improving BB resistance in rice breeding.

## 2. Results

### 2.1. Genotypes and Population Structures of Rice Accessions

The 359 *Japonica* rice accessions were genotyped using a 55K SNP rice array [29]. A total of 28,347 high-quality SNPs were obtained with a missing rate lower than 20% and a minor allele frequency (MAF) of >1%. An ultra-dense SNP genetic map was constructed based on the distribution of SNPs on chromosomes (Figure S1). The SNPs were evenly distributed on each chromosome, with chromosomes 1 and 9 being the longest and shortest, respectively. The number of SNPs on a single chromosome ranged from 963 (chromosome 9) to 3531 (chromosome 1). The average distance between SNPs in the whole genome was 13,128 bp, ranging from 9779 bp (chromosome 11) to 23,639 bp (chromosome 9) (Table S1).

The PCA analysis revealed two major subgroups with 7.26% and 7.01% genetic variation explained by the first two principal components (Figure S2A). The accessions from Korea clustered in a subgroup, while the accessions from Japan and China distributed in two subgroups. Additionally, the heat map of the kinship was consistent with the two major subgroups (Figure S2B).

### 2.2. Resistance of Rice Accessions to Three Xoo Strains

**Figure 1:**
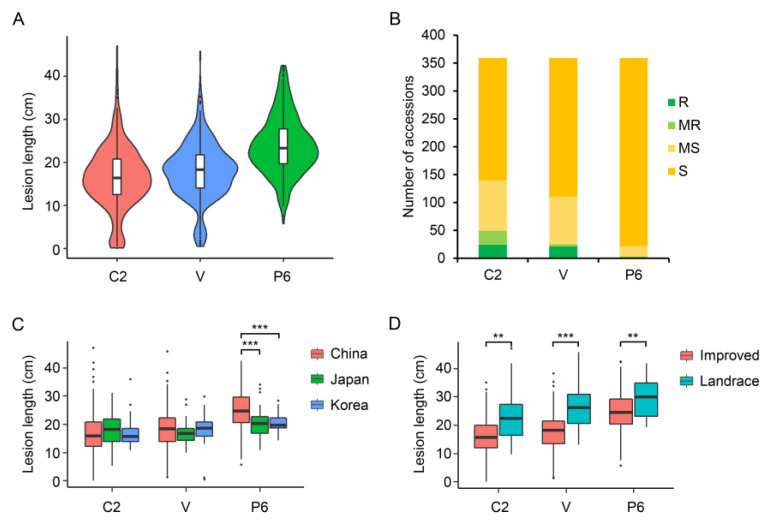
Evaluation of bacterial blight resistance of 359 *Japonica* rice accessions to three *Xoo* strains KS6-6 (C2), GV(V), and PXO99^A^(P6)**.** (**A**), Violin diagram of lesion length among 359 accessions. (**B**), Number of accessions with different resistance levels according to lesion length (LL). Resistant (LL < 5 cm), moderately resistant (5 cm ≤ LL < 10 cm), moderately susceptible (10 cm ≤ LL < 15 cm), and susceptible (LL ≥ 15 cm). (**C**), Boxplots for LLs of 359 accessions from different countries after inoculation with three *Xoo* strains. (**D**), Boxplots for LLs of improved and landrace in 359 accessions after inoculation with three *Xoo* strains. Box edges represent 0.25 and 0.75 quantiles with median values indicated by bold lines. ** and *** refer to significant differences in average LLs among 359 accessions at *p* < 0.01 and 0.001, respectively.

All rice accessions were inoculated with three *Xoo* strains (KS6-6 (C2), GV (V), and PXO99^A^ (P6)) at the primary tillering stage (Table S2). The distribution of lesion length (LL) varied among the 359 accessions (Figure 1A). According to the LL, the majority of accessions were identified as moderately susceptible (10 cm ≤ LL < 15 cm) or susceptible (LL ≥ 15 cm); 86.35%, 93.04%, and 99.16% of the accessions were moderately susceptible or susceptible to strains C2, V, and P6, respectively (Figure 1B). The average LL of improved varieties in China was significantly shorter than that of the landrace varieties (Figure 1D and Figure S3). The varieties from Japan and Korea were more susceptible than the improved varieties in China. For C2 and V, 24 and 21 varieties were highly resistant (LL < 5 cm), respectively. Most of the resistant accessions were from Jiangsu province, China (Table S2). For the virulent strain P6, the average LL was significantly longer in the Chinese accessions than in the Japanese and Korean accessions (Figure 1C). Only three accessions were moderately resistant (5 cm ≤ LL < 10 cm) to P6, and no accessions were highly resistant, suggesting that cultivars conferring resistance to P6 are deficient among the Asian temperate *Japonica* accessions.

### 2.3. Identification of Resistance Loci against BB through GWAS

**Figure 2:**
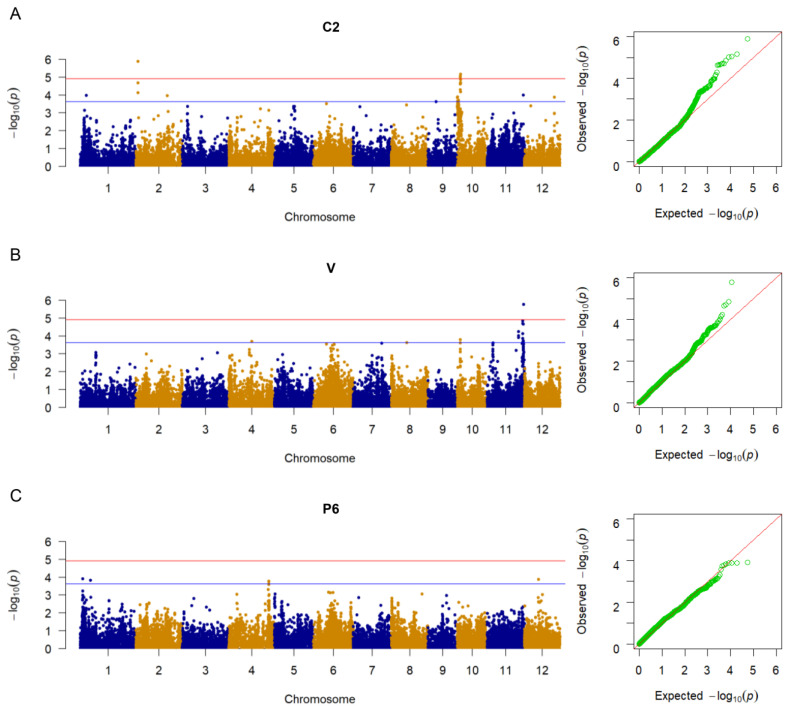
Genome-wide association study (GWAS) of rice resistance to three *Xoo* strains. (**A**–**C**), Manhattan and quantile–quantile plots for GWAS of bacterial blight resistance to C2 (**A**), V (**B**) and P6 (**C**). The horizontal blue line indicates suggestive *p*-value threshold of 2.38 × 10^−4^. The horizontal red line indicates significant *p*-value threshold of 1.19 × 10^−4^.

To detect genome-wide associated loci related to BB resistance against two Chinese *Xoo* strains and one Philippine *Xoo* strain, GWAS was performed based on 28,347 high quality SNPs and phenotypic data. The GWAS was conducted using a mixed linear model in the EMMAX program. On the basis of the independent markers after Bonferroni correction, the suggestive and significant threshold *p*-values were estimated to be 2.38 × 10^−4^ and 1.19 × 10^−5^, respectively. A total of twenty-seven, eighteen, and seven significant SNPs were associated with resistance to C2 (Figure 2A), V (Figure 2B), and P6 (Figure 2C), respectively. The significant SNPs were distributed on all chromosomes except for chromosomes 5, 6, and 7. Based on the calculated LD decay rate of 400 kb (Figure S4), the chromosomal regions for each QTL around the lead SNP were determined. In total, eight QTL containing thirty-three significant SNPs associated with BB resistance to three strains were identified (Figure 2; Table 1). Among the eight QTL, four were co-localized or closely linked to previously mapped genes or QTL in different populations, whereas the other four loci were novel (Table 1).

For C2, four novel QTL on chromosomes 1, 2, and 10 were identified. Notably, there was a steep peak of resistance-related SNPs in *qBBC2-10.2*. For V, 14 significantly associated SNPs were identified on chromosome 11. Based on linkage disequilibrium (LD) analysis, this region was divided into three QTL (*qBBV-11.1*, *qBBV-11.2*, and *qBBV-11.3*). For P6, only one resistance locus, *qBBP6-4*, defined by two significant SNPs within the region 30,856,230–31,068,491 bp, was identified on chromosome 4. This QTL was co-localized with the previously mapped QTL associated with resistance to *Xoo* strain GD1358 from China [20].

### 2.4. Candidate Gene Prediction

In the 359 *Japonica* rice accessions, four new QTL associated with resistance to C2 were identified: *qBBC2-1*, *qBBC2-2*, *qBBC2-10.1*, and *qBBC2-10.2*. Within *qBBC2-1* localized in the region of 4,747,337–4,961,779 bp on chromosome 1, *LOC_Os01g09370* with the lead SNP rs1_4762032 was predicted as a candidate gene. This gene encodes a protein of the same family as XA21 binding protein 3, both encoding ankyrin repeat domain-containing proteins [33]. In *qBBC2-2* (1,614,380–1,778,296 bp on chromosome 2), *LOC_Os02g03950* with the significant SNP rs2_1683645 was predicted as a candidate gene. This gene encodes a zinc finger nucleic acid-binding protein. The receptor kinase-like gene *LOC_Os10g01060* was predicted as a candidate gene based on two significant missense SNPs, rs10_67342 and rs10_70306, in *qBBC2-10.1* (58,063–850,339 bp on chromosome 10). *LOC_Os10g01060* had two major haplotypes according to the two missense SNPs. There was a significant difference in LL between the two haplotypes, and Hap1 conferred a higher level of resistance to C2 (Figure S5). Additionally, *qBBC2-10.2* (2,375,502–2,569,504 bp on chromosome10) contained an F-box gene cluster comprising *LOC_Os10g04900* (*OsFBX364*) and *LOC_Os10g05000* (*OsFBX366*), which was predicted to be associated with resistance to C2 (Figure S6). Based on the significant SNP rs10_2387770 in *LOC_Os10g04900*, two major haplotypes were identified with significantly different LLs (Figure S6B). Two major haplotypes of *LOC_Os10g05000* with significantly different LLs were also identified using the missense SNP rs10_2439188 (*p* = 1.98 × 10^−5^) (Figure S6C).

For V, a steep peak of significant SNPs was detected on chromosome 11, including three QTL: *qBBV-11.1*, *qBBV-11.2*, and *qBBV-11.3.* In *qBBV-11.1* (27,648,838–27,675,916 bp), two significant SNPs (rs11_27673200 and rs11_27675916) were located in *LOC_Os11g45740*, encoding a MYB transcription factor. Two major haplotypes of *LOC_Os11g45740* were distinguished based on the nonsynonymous SNPs (Figure S7A), and the average LL of Hap2 was significantly shorter than that of Hap1 after inoculation with V (Figure S7B). In *qBBV-11.2* (27,987,854–28,010,037 bp), the candidate gene *LOC_Os11g46210* contained two significant SNPs: rs11_27988169 and rs11_27988643. Four major haplotypes of *LOC_Os11g46210* were detected using eight missense SNPs in its coding region (Figure S7C), and the average LL of Hap1 was significantly shorter than those of Hap2 and Hap3 after inoculation with V (Figure S7D).

**Figure 3:**
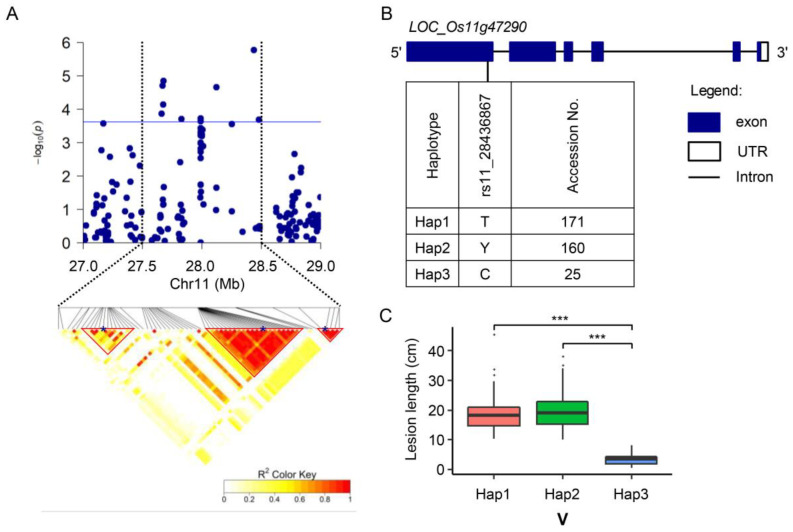
Analysis of associated region of *qBBV-11.3* and haplotype analysis of *LOC_Os11g47290*. (**A**), Local Manhattan plot (top) and linkage disequilibrium heat map (bottom) for region surrounding the lead SNP. The position of lead SNP in each QTL is marked with blue asterisk. (**B**), Gene structure and haplotype analysis of *LOC_Os11g47290*. Haplotypes with fewer than 10 accessions are not shown. (**C**), Lesion length (LL) of accessions with different haplotypes of *LOC_Os11g47290* inoculated with *Xoo* strain V. *** indicates significant difference in average LLs among 359 accessions at *p* < 0.001.

In *qBBV-11.3*, two significant SNPs were closely associated with resistance to V (Figure 2B), and the lead SNP with *p*-value of 1.69 × 10^−6^ (rs11_28436867) was located in *LOC_Os11g47290*, which encodes a leucine-rich repeat receptor-like kinase (LRR-RLK) (Figure 3A). After performing haplotype analysis using the significant SNP associated with BB resistance, the accessions were divided into three major haplotypes (Figure 3B). The average LL of Hap3 was significantly shorter than those of Hap1 and Hap2 after inoculation with V (Figure 3C). *Xa3/Xa26* was also located within the *qBBV-11.3* region (28,436,867–28,484,909 bp), but no SNPs were detected in this gene in the 55K SNP Array.

For P6, only *qBBP6-4* was identified in the 30,856,230–31,068,491 bp interval on chromosome 4. *LOC_Os04g52200* and *LOC_Os04g52210* contained the significant SNPs rs4_31010527 and rs4_31023804, respectively. *LOC_Os04g52200* encodes an RNA recognition motif-containing protein, which includes five RNA-binding domains. *LOC_Os04g52210* encodes a terpene synthase with terpenoid cyclase/protein prenyltransferase domains. In addition, a significant SNP rs4_30967438 was located in the 3′ downstream region of the gene *LOC_Os04g52130,* which was reported to be associated with BB resistance [34].

### 2.5. Involvement of LOC_Os11g47290 Strain-Specific Resistance to BB

**Figure 4:**
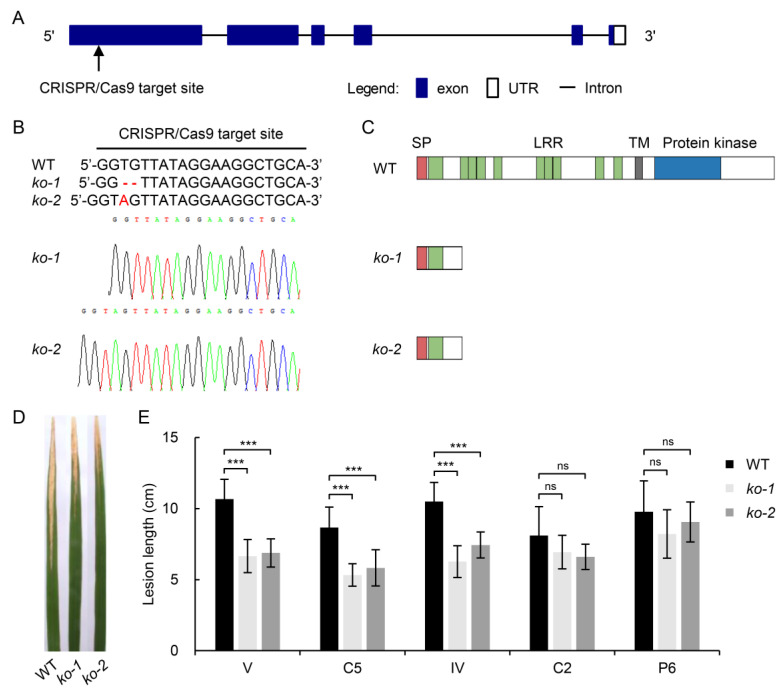
Functional characterization of *LOC_Os11g47290* based on CRISPR/Cas9-mediated genome editing. (**A**), Schematic map of gRNA target sites on genomic regions of *LOC_Os11g47290*. (**B**), Mutation site of knockout mutants generated by CRISPR/Cas9 genome editing in Nipponbare. (**C**), Schematic diagrams of LOC_Os11g47290 and truncated proteins in knockout mutants. SP, signal peptide; LRR, leucine-rich repeat region; TM, transmembrane domain. (**D**), Phenotypes of wild type (WT) and *ko-1* and *ko-2* mutants inoculated with *Xoo* strain V. (**E**), Lesion lengths (LLs) 14 days after inoculation with five *Xoo* strains (V, C5, IV, C2, and P6). Values are mean ± SD (*n* = 10). *** *p* < 0.001 (Student’s *t*-test).

The LRR-RLKs are a ubiquitous gene family in higher plants. Members of this family encode receptors of extracellular signals that play critical roles in development and defense [35,36,37]. In *qBBV-11.3*, *LOC_Os11g47290*, encoding an LRR-RLK, was predicted to be associated with resistance to the strongly virulent *Xoo* strain V. SMART analysis (http://smart.embl-heidelberg.de/, accessed on 6 March 2020) indicated that LOC_Os11g47290 contains a signal peptide domain, 10 LRR domains, a transmembrane domain (TM), and a protein kinase domain (Figure 4C). *LOC_Os11g47290* had three major alleles at the significant SNP in the 359 *Japonica* rice accessions, with 92.2% of accessions belonging to susceptible Hap1 and Hap2 and with 6.96% of accessions belonging to the resistant Hap3 (Figure 3B).

To investigate whether *LOC_Os11g47290* is involved in BB resistance in rice, knockout mutants of *Japonica* cultivar Nipponbare (Hap1) were constructed using the CRISPR/Cas9 gene editing system. Two homozygous mutants were identified among 30 T_0_ transgenic lines (Figure 4A). Mutant *ko-1* had a 2-bp deletion at the target site, and mutant *ko-2* had a 1-bp insertion at the target site (Figure 4B). In both *ko-1* and *ko-2*, editing *LOC_Os11g47290* had caused frameshift mutation or premature termination of protein translation (Figure 4C). The LLs of *ko-1* and *ko-2* were significantly shorter than that of wild-type after inoculation with V (Figure 4D,E). To evaluate the resistance spectrum, the mutants were further evaluated with four other *Xoo* strains, namely C2, P6, and two strongly virulent *Xoo* strains from South China: C5 and IV. The latter two strains can breakdown resistance mediated by *Xa4* or *Xa21*. The results showed that *ko-1* and *ko-2* exhibited enhanced resistance to C5 and IV, whereas their resistance to C2 and P6 was not significantly different from that of wild type (Figure 4E). These results indicate that *LOC_Os11g47290* is related to strain-specific resistance in rice.

## 3. Discussion

### 3.1. Variation in Resistance of the Temperate Japonica Rice Accessions

Up to now, the BB resistance of *indica* accessions were screened [11,20,21], whereas, there was less on the exploitation of resistant germplasm in *Japonica* panels. In this study, we evaluated the resistance of 359 Asian temperate *Japonica* accessions from China, Japan, and Korea to two Chinese representative *Xoo* strains (C2 and V) and one Philippine *Xoo* strain (P6). C2 is a representative strain of pathotype II from the Yangtze River basin in China [38]; V is a virulent strain found in Guangdong Province, China, that can overcome the resistance mediated by *Xa21* [39], and P6 is a strongly virulent *Xoo* strain from the Philippines. Some rice accessions resistant to the three *Xoo* strains were found in this collection of *Japonica* rice germplasm (Table S2). For P6, there were no highly resistant cultivars among the 359 accessions, and only three Chinese cultivars, Yanggeng 282, Fu 105, and Yangeng 218, showed moderate resistance. Of them, Yanggeng 282 was also resistant to both C2 and V. Moreover, six Japanese varieties were moderately resistant to C2, and two Korean varieties were highly resistant to V. The cultivars showing high resistance to C2 and V were mainly from Jiangsu Province, in China. No varieties from the rice-growing region of Northeast China (Liaoning, Jilin, and Helongjiang) exhibited resistance to BB, suggesting that the BB resistance of cultivars in this region has not been under natural or artificial selection.

### 3.2. QTL and Candidate Genes Associated with BB Resistance

Eight loci containing 33 significant SNPs associated with BB resistance were identified in the 359 *Japonica* rice accessions (Figure 2, Table 1). Of them, four QTL overlapped with previously reported BB resistance QTL or genes (Table 1). *qBBP6-4* associated with resistance to P6 overlapped with *qC5-5* [20]. *qBBV-11.1* containing the *R* genes *Xa35(t)* [6] and *Xa36(t)* [6] overlapped with L11 [17], *qC4-11* [20], *qBB11.4* [11], and *qBLB11:1* [30]. *qBBV-11.2* containing the *R* genes *Xa22*(t) [6], *Xa35(t)* [6], *Xa36(t)* [6], *xa44(t)* [6], and *Xa47* [7] overlapped with *qC5-11.1* [20], *qBB11.5* [11], *QBbr11-2* [31], and *qBLB11:1* [30]. *qBBV-11.3* containing the *R* genes *Xa3/Xa26* [6] and *Xa43(t)* [32] overlapped with *qC5-11.2* [20], *qBB11.6* [11], and *qBLB11:1* [30]. Four of the eight QTL detected in the 359 rice accessions were novel loci related to *Xoo* resistance. One novel locus was located on chromosome 1; one was located on chromosome 2, and two were located on chromosome 10. On chromosome 2, only *xa24(t)* and three QTL have been identified previously [6,17,31], and none of them identified in this study overlapped with *qBBC2-2*. So far, no BB *R* genes or QTL have been identified on chromosome 10.

The cloned BB *R* genes can be classified into five categories according to the functions of their encoded proteins: genes encoding NLRs (*Xa1*, *Xa2/Xa31(t)*, *Xa14*, *Xa45(t)*, and *Xa47*), genes encoding receptor-like kinases (*Xa3/Xa26*, *Xa4*, and *Xa21*), genes encoding sugar transporter (SWEET) proteins (*xa13/ossweet11*, *xa25/ossweet13*, and *xa41/ossweet24*), executor *R* genes (*Xa7*, *Xa10*, *Xa23*, and *Xa27*), and other genes (*xa5*) [6]. Among the candidate genes identified in this study, *LOC_Os11g46210* in *qBBV-11.2* encodes an NLR, and *LOC_Os10g01060* and *LOC_Os11g47290* in *qBBC2-10.1* and *qBBV-11.3* both encode receptor kinase-like proteins.

Other than *R* genes, some genes encoding regulatory factors, such as the WRKY-type transcription factors WRKY13 and WRKY45-2, the MYB transcription factor OsMYB63, and the CCCH-type zinc finger nucleic acid-binding protein C3H12, have been reported to be involved in host pathogen species-non-specific broad-spectrum resistance [40,41,42]. Thus, strategies targeting these genes may be an effective and sustainable approach to control disease prevalence [43,44]. Based on the lead SNPs in each QTL, the predicted candidate genes included *LOC_Os02g03950* and *LOC_Os10g05230* encoding zinc finger nucleic acid-proteins in *qBBC2-2* and *qBBC2-10.2*, respectively, and *LOC_Os11g45740* encoding a MYB transcription factor in *qBBV-11.1*. In *qBBC2-10.2*, we identified an F-box gene cluster composed of *LOC_Os10g04900* (*OsFBX364*) and *LOC_Os10g05000* (*OsFBX366*). Some F-box genes might be involved in regulating disease resistance in plants. For example, *ZmFBL41* confers resistance to banded leaf and sheath blight in maize [45]. *SON1* in *Arabidopsis* is involved in defense response to the bacterial pathogen *Pseudomonas syringae* pv *tomato* [46]. It was reported that the plants overexpressing *OsTPS19*, which encodes a terpene synthase, enhance blast resistance in rice [47]. In this study, *LOC_Os04g52210* encoding terpene synthase in *qBBP6-4* was identified as a candidate gene associated with BB resistance. The functions of these genes in response to BB need to be further validated in further research.

### 3.3. Improving BB Resistance in Rice through Editing LOC_Os11g47290

The RLKs are a distinct group of important receptors composed of an extracellular binding domain, a transmembrane domain, and an intracellular kinase domain. They exert critical roles in plant immunity, growth, and development [37,48]. Some LRR-RLKs are crucial components of plant immune responses [49]. Among the cloned BB *R* genes in rice, *Xa3/Xa26* and *Xa21* encode LRR-RLKs [6]. OsSERK2 positively regulates immunity mediated by XA21 and XA3 immune receptors and is an LRR-RLK similar to XA21 [50]. Here, we found that the LRR-RLK gene *LOC_Os11g47290* associated with BB resistance in *qBBV-11.3* had three alleles at the significant SNP (rs11_28436867) (Figure 3). Because most accessions carried susceptible haplotypes, we generated knockout mutants of Nipponbare with the susceptible haplotype using the CRISPR/Cas9 gene editing system (Figure 4). The knockout lines showed enhanced resistance to the Chinese virulent strains IV, V, and C5. These results provide evidence that editing of *LOC_Os11g47290* using the CRISPR/Cas9 system is a promising strategy for improving the BB resistance of rice.

In recent decades, strategies to improve rice BB resistance have relied on *R* genes. With the development of gene editing techniques, some efforts have been made to improve the BB resistance of rice by editing the promoters of susceptibility genes, including SWEET genes and the recessive allele of the *R* gene *xa13* [26,27]. Notably, genome-edited SWEET promoters may endow rice lines with robust, broad-spectrum resistance to BB [24,51]. However, this type of resistance to BB conferred by changes in promoter sequences will not prevent adaptation of the pathogen, and the durability of this approach will depend on the ability of *Xoo* populations to adapt to recessive resistance alleles [24]. Therefore, it is necessary to identify more targets for genome editing for disease resistance. Compared with traditional genetic mapping using bi-parental populations, GWAS can detect more alleles at one locus by exploiting larger numbers of historical recombination events in varieties with more genetic diversity [52]. The predicted candidate genes associated with BB resistance and their haplotypes identified in this study represent useful information for cloning BB resistance genes and improving BB resistance in rice-breeding programs.

## 4. Materials and Methods

### 4.1. Plant Materials and Growth Conditions

A panel of 359 rice accessions derived from East Asian temperate *Japonica* accessions were used to perform GWAS. Among them, 276 accessions (22 landraces, 254 improved varieties) were from China; 51 accessions were from Japan, and 32 accessions were from Korea (Table S2). The seeds were soaked in ethylicin solution and sown in a seedling nursery house. The 30-day-old seedlings of each accession were transplanted into paddy fields at experimental farms of the Institute of Crop Sciences, Chinese Academy of Agricultural Sciences, Beijing, China. Each of the rice accessions was planted in a 3-row plot with seven plants per row at a spacing of 20 × 17 cm^2^. The field planting followed a randomized complete block design with two experimental replicates. All plants were managed under standard cultivation practices.

### 4.2. Xoo Strains and Artificial Inoculation

The representative *Xoo* strains KS6-6 (C2) and GV (V) from China and PXO99^A^ (P6) from the Philippines were used for evaluation of BB resistance of the 359 rice accessions. C2, V, P6, GD1358 (C5), and IV were used to evaluate the resistance of knockout mutants of *LOC_Os11g47290*. Each *Xoo* strain was incubated on peptone sucrose agar at 30 °C for 2 days, and the inoculum was prepared by suspending the bacterial mass in sterile water at a concentration of 10^8^ cells/mL. The rice plants were inoculated by the leaf-clipping method at the tillering stage, 30 days after transplanting [53]. For each *Xoo* strain, each accession or transgenic line was inoculated for five plants by clipping at least five leaves per plant. The LL of each plant was measured 3 weeks after inoculation, on three leaves per inoculated plant in each experimental replicate. Based on the LL data, the rice accessions were determined to be resistant (LL < 5 cm), moderately resistant (5 cm ≤ LL < 10 cm), moderately susceptible (10 cm ≤ LL < 15 cm), or susceptible (LL ≥ 15 cm) [17].

### 4.3. Genotyping with the 55K SNP Array

Genomic DNA was isolated from each of the 359 accessions using the cetyl trimethyl ammonium bromide (CTAB) method [54]. Genotyping analysis was performed using a customized rice 55K SNP array, which contains 54,837 SNPs screened from the 3K Rice Genome Project [19,29]. Chip genotyping was conducted by CapitalBio Technology (Beijing, China) according to the Affymetrix Axiom^®^ 2.0 assay protocol.

### 4.4. Population Structure Analysis

Using the linkage disequilibrium (LD) pruning tool of PLINK 1.9 [55], we obtained independent SNPs with a genotype missing rate ≤20% and minor allele frequency ≥1% according to the settings “indep-pairwise 50 10 0.5”. A principal component analysis (PCA) was performed using smart PCA in the EIGENSOFT program [56]. Genetic relationships between rice accessions were estimated using the Balding–Nichols method, and a kinship matrix was generated [57].

### 4.5. Genome-Wide Association Analysis

To minimize the effect of false positives, 55K SNPs with the missing rate ≥ 20% and MAF ≤ 1% were removed by PLINK 1.9 software [55]. Finally, a total of 28,347 SNPs in the 359 rice accessions and the average LL of each accession were used to carry out GWAS. The Efficient Mixed-Model Association eXpedited (EMMAX) was used to perform GWAS based on the SNP genotypes and LLs on plants inoculated with the three *Xoo* strains using a mixed linear model linear (MLM), principal component analysis (PCA), and kinship [58]. The effective number of independent markers (N = 4193) was calculated with GEC software [59]. The suggestive (1/N) and significant (0.05/N) *p*-value thresholds were set as 2.38 × 10^−4^ and 1.19 × 10^−5^ to control the genome-wide type 1 error rate [60]. Manhattan and quantile–quantile (Q-Q) plots were created using the R package “qqman” [61].

### 4.6. QTL Identification and Analysis of Candidate Genes

According to LD decay, the region 400 kb upstream and downstream of a significant SNP was classified as an LD block. An LD block containing more than two significant SNPs was defined as a candidate QTL [20,62,63]. In each QTL, the SNP with minimum *p*-value was considered as the lead SNP [64]. The LDs between SNPs were evaluated using squared Pearson’s correlation coefficient (*r2*) calculated with the R package “genetics”. The local LD interval was estimated from the continuous region closely linked to the lead SNP (*r2* ≥ 0.6) [65]. The LD heatmap was constructed using the R package “LD heatmap” [55,66].

For candidate genes in each QTL, a haplotype analysis was performed using the 55K SNP array of 359 rice accessions. Haplotypes containing more than 10 rice accessions were identified as major haplotypes. To explain variations in phenotype, significant differences in the LLs of the major haplotypes of candidate genes were determined by ANOVA. The most likely candidate genes were predicted according to significant differences in the LLs of their haplotypes for each QTL.

### 4.7. Vector Construction and Rice Transformation

CRISPR/Cas9 gene editing was used to generate knock-out mutants of *LOC_Os11g47290*. As described by Ma et al. [67], one guide RNA was designed to target the exon of *LOC_Os11g47290* using the web-based software CRISPR-GE (http://skl.scau.edu.cn/, accessed on 10 March 2020) [68]. A 20 bp gene-specific guide of RNA sequences targeting *LOC_Os11g47290* was synthesized, annealed, and ligated into pYLgRNA-U6a and then inserted into the pYLCRISPR/Cas9PUbi-H vector. The CRISPR/Cas9 vector was transformed into the *Japonica* rice cultivar Nipponbare by *Agrobacterium tumefaciens*-mediated transformation [69]. The mutation types were identified by PCR-based sequencing. The resistance of T_3_ generation homozygous mutants and WT were evaluated in the field of screening house. The primers used in this study are shown in Table S3.

## Figures and Tables

**Table 1:** Genetic loci associated with bacterial blight resistance through genome wide association study.

QTL Name ^a^	*Xoo* Strains	Chromosome	LD Block Interval (bp)	Number of Significant SNP	Lead SNP	*p*-Value	PVE ^b^ (%)	Effect	Reported QTL/Genes
*qBBC2-1*	C2	1	4,747,337–4,961,779	2	rs1_4762032	1.03 × 10^−4^	3.8	6.01	Novel
*qBBC2-2*	C2	2	1,614,380–1,778,296	3	rs2_1651016	1.28 × 10^−6^	6.1	−6.08	Novel
*qBBC2-10.1*	C2	10	58,063–850,339	6	rs10_187309	1.26 × 10^−4^	3.5	−3.36	Novel
*qBBC2-10.2*	C2	10	2,375,502–2,569,504	10	rs10_2508049	6.82 × 10^−6^	4.4	−4.51	Novel
*qBBV-11.1*	V	11	27,648,838–27,675,916	4	rs11_27660271	1.36 × 10^−4^	4.6	3.80	*Xa35(t)* [6], *Xa36(t)* [6], L11 [17], *qC4-11* [20], *qBB11.4* [11], *qBLB11:1* [30]
*qBBV-11.2*	V	11	27,987,854–28,010,037	2	rs11_27988643	1.88 × 10^−4^	4.0	−1.59	*Xa22(t)* [6], *Xa35(t)* [6], *Xa36(t)* [6], *xa44(t)* [6], *Xa47* [7], *qC5-11.1* [20], *qBB11.5* [11], *QBbr11-2* [31], *qBLB11:1* [30]
*qBBV-11.3*	V	11	28,436,867–28,484,909	2	rs11_28436867	1.69 × 10^−6^	22.8	3.23	*Xa3/Xa26* [6], *Xa43(t)* [32], *qC5-11.2* [20], *qBB11.6* [11], *qBLB11:1* [30]
*qBBP6-4*	P6	4	30,856,230–31,068,491	2	rs4_31010527	1.70 × 10^−4^	3.8	2.62	*qC5-4* [20]

^a^ *qBBX-N* indicates QTL located on chromosome N conferring bacterial blight resistance to X. ^b^ PVE, phenotypic variation explained.

## Data Availability

The data supporting the findings of this study are available within the article and its Supplementary Materials.

## Supplementary Materials

The supporting information can be downloaded at: https://www.mdpi.com/article/10.3390/ijms24108810/s1.
