# Supplementary material for: Genome-Wide Association Study Identifies Resistance Loci for Bacterial Blight in a Collection of Asian Temperate Japonica Rice Germplasm
Source: Int J Mol Sci. 2023 May 16;24(10):8810. doi: 10.3390/ijms24108810 (PMC10218538; doi:10.3390/ijms24108810)
Supplement: Supplementary file 1 [file ijms-24-08810-s001.zip › Figures S1-S7.pdf]

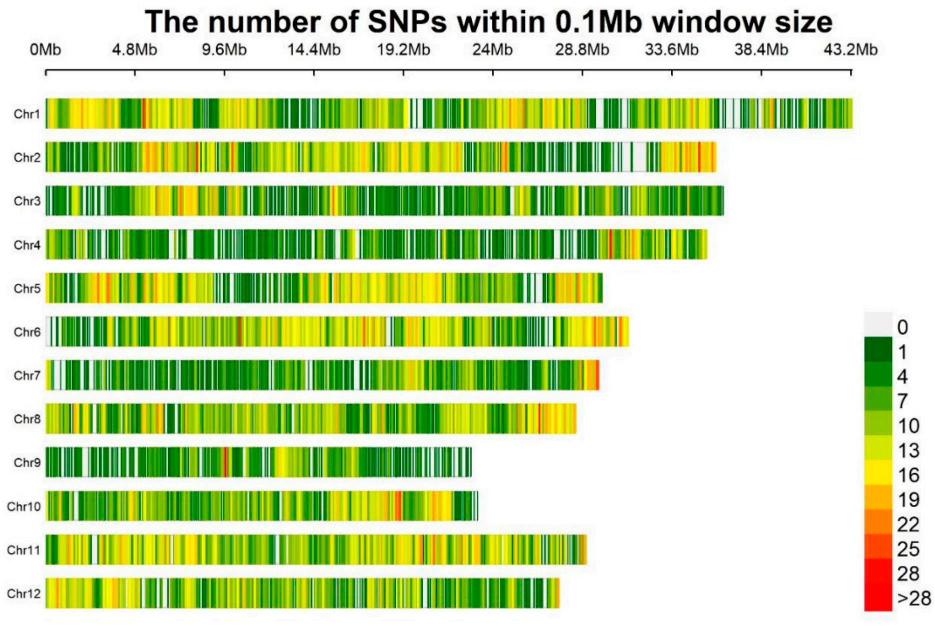

Figure S1. SNP density map for rice chromosomes.

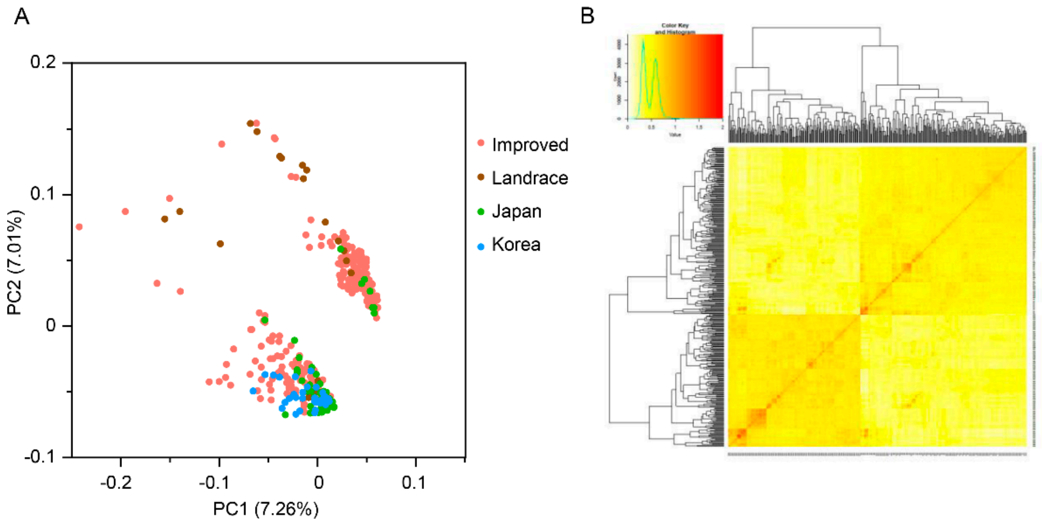

Figure S2. Population structure of 359 rice accessions.

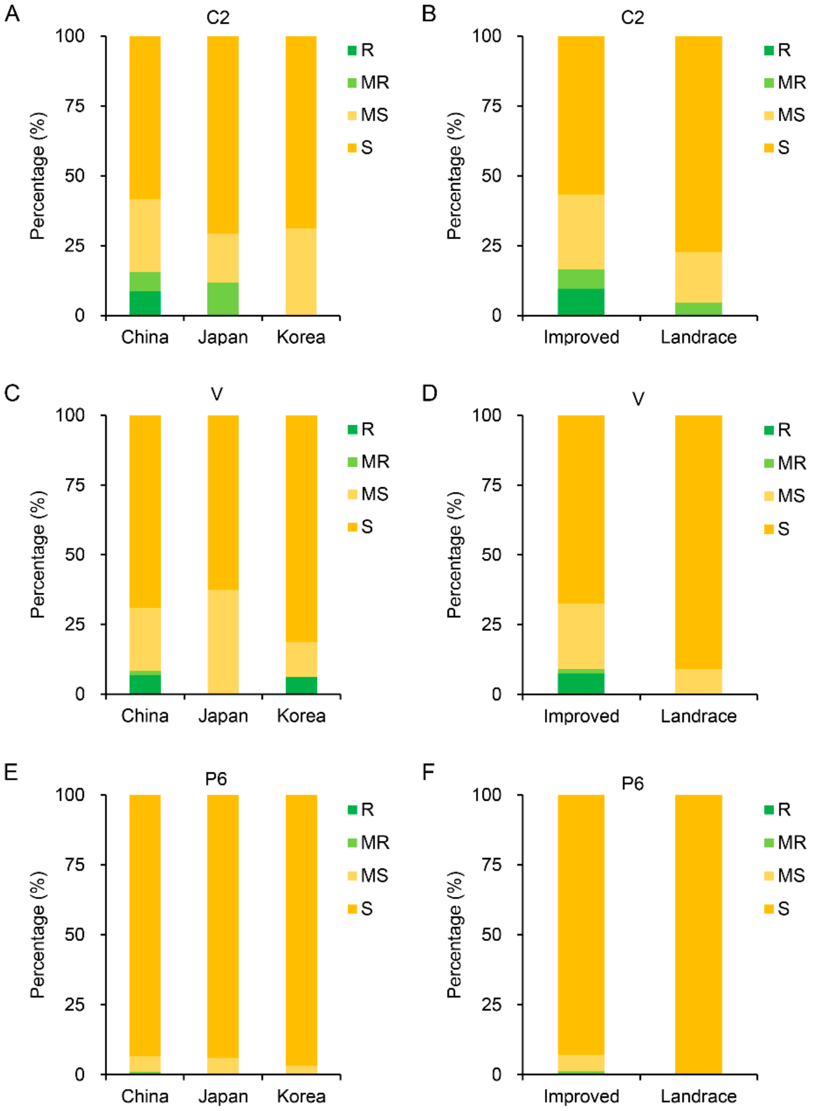

Figure S3. Boxplots for LLs of 359 accessions inoculated with three *Xoo* strains.

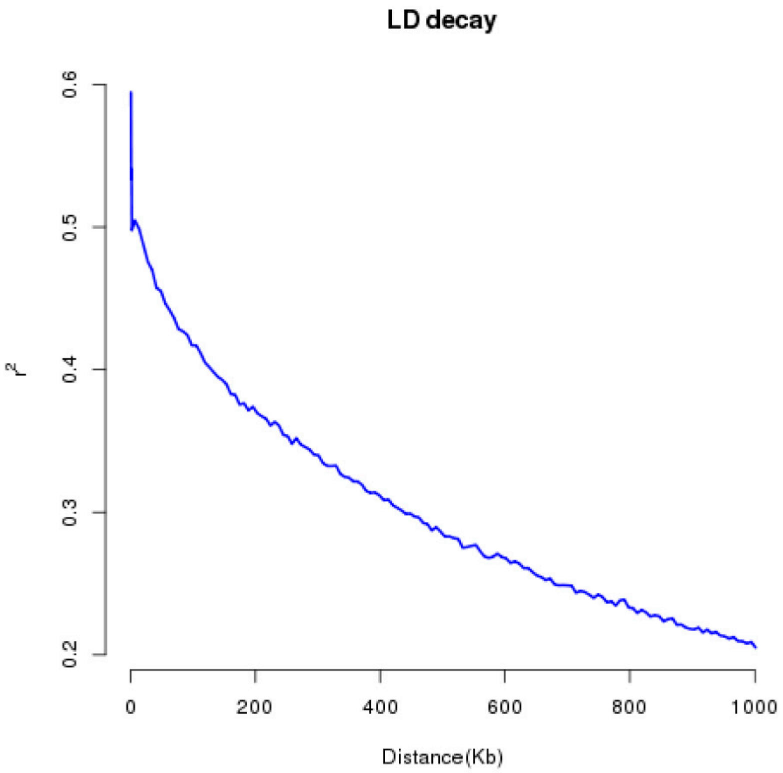

Figure S4. LD (*r2*) decay analysis of 359 *Japonica* rice accessions.

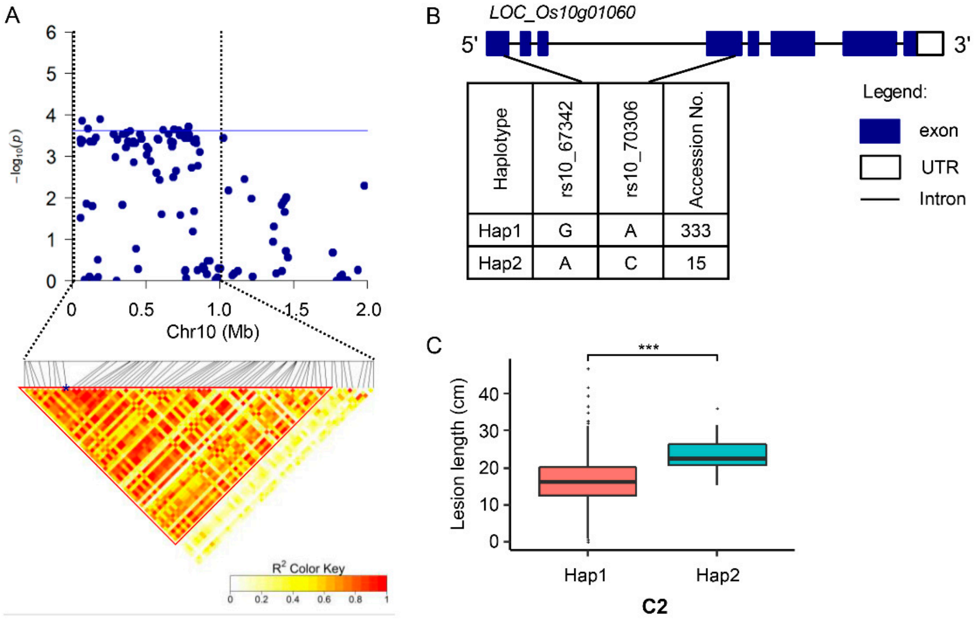

Figure S5. Analysis of associated region of *qBBC2-10.1* and haplotype analysis of *LOC_Os10g01060*.

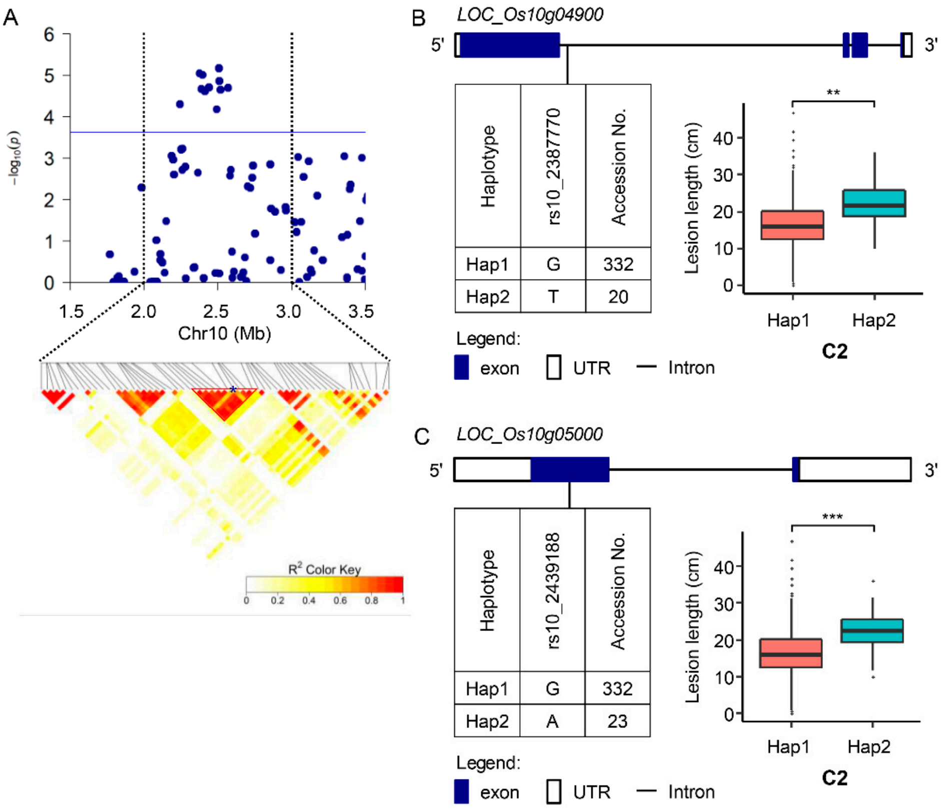

Figure S6. Analysis of associated region of *qBBC2-10.2* and haplotype analysis of *LOC_Os10g04900* and *LOC_Os10g05000*.

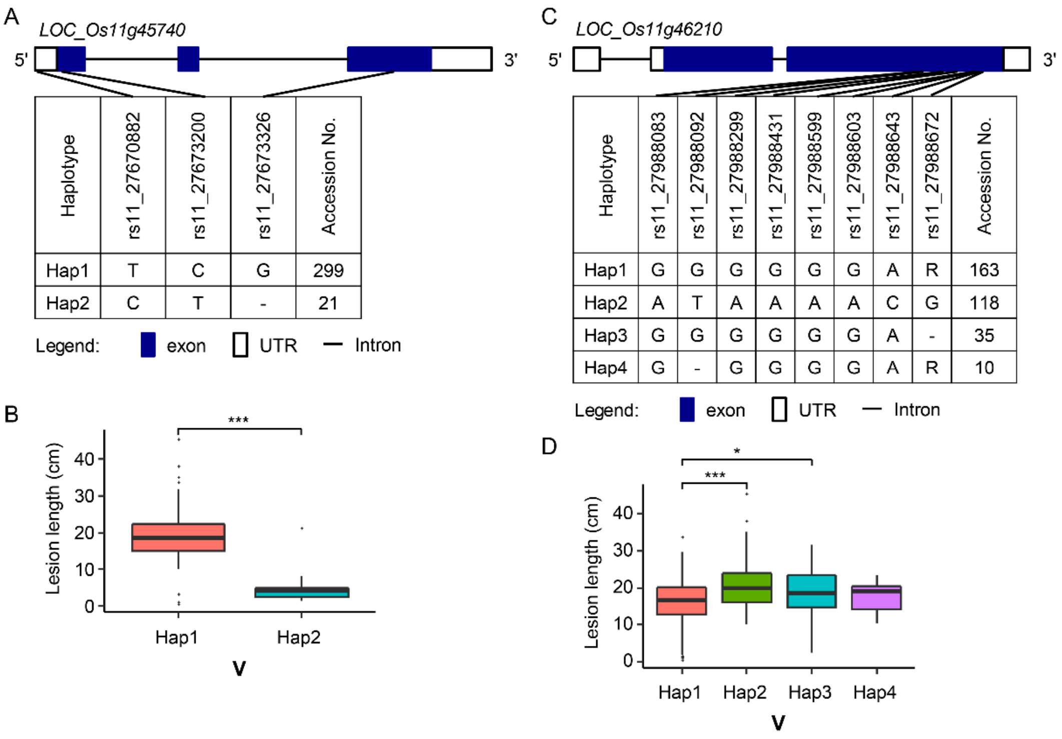

Figure S7. Gene structure and haplotype analysis of *LOC_Os11g45740* and *LOC_Os11g46210*.
